# Variability in Cross‐Domain Risk Perception among Smallholder Farmers in Mali by Gender and Other Demographic and Attitudinal Characteristics

**DOI:** 10.1111/risa.12976

**Published:** 2018-02-15

**Authors:** Alison C. Cullen, C. Leigh Anderson, Pierre Biscaye, Travis W. Reynolds

**Affiliations:** ^1^ Daniel J. Evans School of Public Policy and Governance University of Washington Seattle WA USA; ^2^ Environmental Studies Program Colby College Waterville ME USA

**Keywords:** Development, gender, Mali, risk perception, smallholder farmers

## Abstract

Previous research has shown that men and women, on average, have different risk attitudes and may therefore see different value propositions in response to new opportunities. We use data from smallholder farm households in Mali to test whether risk perceptions differ by gender and across domains. We model this potential association across six risks (work injury, extreme weather, community relationships, debt, lack of buyers, and conflict) while controlling for demographic and attitudinal characteristics. Factor analysis highlights extreme weather and conflict as eliciting the most distinct patterns of participant response. Regression analysis for Mali as a whole reveals an association between gender and risk perception, with women expressing more concern except in the extreme weather domain; however, the association with gender is largely absent when models control for geographic region. We also find lower risk perception associated with an individualistic and/or fatalistic worldview, a risk‐tolerant outlook, and optimism about the future, while education, better health, a social orientation, self‐efficacy, and access to information are generally associated with more frequent worry—with some inconsistency. Income, wealth, and time poverty exhibit complex associations with perception of risk. Understanding whether and how men's and women's risk preferences differ, and identifying other dominant predictors such as geographic region and worldview, could help development organizations to shape risk mitigation interventions to increase the likelihood of adoption, and to avoid inadvertently making certain subpopulations worse off by increasing the potential for negative outcomes.

## 1. INTRODUCTION

Much of the considerable body of published scholarship exploring the association between gender and risk perception has been conducted in the United States and Europe, which differ substantially from the rural smallholder agricultural contexts typical of many low‐income countries. Research in rural low‐income contexts is limited to several seminal studies on pastoralists in Eastern Africa queried about their perception of livelihood risks.^1^, ^2^, ^3^ This work highlights the primacy of perceived risks to food and water availability in rural people's risk perceptions, and despite challenges with data structure, identifies some gender basis to variation in perception. This variation is attributed to gendered roles and responsibilities rather than inherent attitudes, consistent with earlier work in the United States and Europe, though a departure from other findings.^4^, ^5^, ^6^, ^7^ Barrett *et al*.^2^ specifically emphasize the need to recognize heterogeneity in risk assessments resulting from “even modest variation in agroclimatic conditions, historical experiences, or differentiation” with demographic factors, which characterize smallholder farming contexts. Further, these researchers stress the need to disaggregate heterogeneous perceptions of risk across subpopulations in order to inform efforts aimed at reducing risk. These stated research gaps are the motivation for our work.

Recent work on farmer populations explores biophysical, sociodemographic, psychological, and social factors shown to influence risk perceptions and resultant decision making^8^ and the perhaps related tendency to risk aversion by farmers in response to opportunities to update methods.^9^ Specifically, predictors of farmers’ risk perceptions include exposure to risk factors (affected by geography, economic status, and access to mitigation strategies), knowledge and understanding of risk sources (affected by experience, education, culture), and individual and community attitudes toward risk. We further note that much of the existing scholarship on farmers focuses on individuals (often male) responding on behalf of households, but that in many smallholder farming households adults of both genders are farmers, and thus farm management decisions have both a household‐level component (a product of male and female household members’ negotiations) and a plot‐level component (which may be entirely male, entirely female, or mixed).

Despite these important differences, there is little research in rural low‐income contexts since the early work of Smith *et al*.,^1^ Barrett *et al*.,^2^ and Quinn *et al*.^3^ comparing risk perceptions across genders among pastoralists, and across different risks, and less still pertaining to smallholder farmers.^10^ Progress has been limited by data sets insufficiently large to examine heterogeneity in risk perception by domain, and across individual characteristics. Moreover, previous work has relied on data collection approaches that required assumptions of consistency between individuals within groups surveyed in terms of the specific risks they perceive, and the amount of concern that they partition among these risks, for statistical tractability. And no previous research on smallholder farmers has conducted a multivariate analysis controlling for the broader set of factors found to be associated with risk perception in the literature. Thus a full accounting of the role of gender has been elusive.

Risk research has long highlighted differences between the perceptions of those at risk (citizens and/or the public), and external assessments such as by experts, governments, or development organizations, which could explain in part the low demand for development programs by farmers.^11^, ^12^, ^13^, ^14^, ^15^, ^16^ However, we observe that even where interventions reasonably identify the risks to individuals of maintaining the status quo, few organizations explicitly recognize the countervailing risks of new technology or program adoption.^14^ An improved understanding of risk perception, and specifically gender‐based differences in low‐income farm households, could thus inform theory surrounding intrahousehold decision making, and household willingness to take risks and engage in new activities, possibly influencing the likelihood of successful development strategies. Understanding if, and how, risk perceptions differ by gender, geographic region, and other significant predictors might also support better intervention designs targeting specific subpopulations. Moreover, to the extent that risk preferences and decision‐making authority are not evenly distributed within a community or even within a given household, some individuals (e.g., husbands or males in general) may be making choices on behalf of others (e.g., wives or females in general) who see those choices as too risky (and thus undesirable). In order to ensure that broad‐based interventions do not inadvertently make certain subpopulations worse off, it is important to account for individual differences in risk perception related to these programs.

In this article we explore the question: Do risk attitudes and perceptions differ systematically between male and female smallholder farmers in Mali across different risk domains? We explore six domains of potential risk including health/injury, climate and extreme weather, social relationships, financial position, livelihood stability, and conflict/security. We examine patterns in risk perception across domains for all individuals, and further where our data reveal systematic deviations by gender, we analyze other factors potentially associated with these differences, such as age, education, health, wealth, geographic region, time poverty, number of children, social orientation, access to information, worldview, optimism about the future, and beliefs about self‐efficacy.

The remainder of this article is organized as follows. First, we summarize the literature on differences in men's and women's risk perceptions in general, and in rural low‐income contexts specifically. Next we describe our data set, sampling approach, and survey population. Then, we develop our basic analytic and statistical methodology. Finally, we present the results of our statistical analysis. We apply dimension reduction (factor analysis) across the set of risk domains in order to distinguish patterns of risk perception in individual responses, and also potential groupings of risk domains for which individuals tend to express similar perceptions. We then present multivariate regression models predicting risk perceptions across domains as a function of household and individual characteristics, including gender, followed by robustness analyses that explore the role of geographic location (with region as a proxy). We end with a discussion of our results and overall conclusions.

## 2. DIFFERENCES IN MEN'S AND WOMEN'S RISK PERCEPTIONS

There is a rich literature exploring potential differences between men's and women's risk perceptions that seeks to explain the complex and heterogeneous patterns that emerge, as well as the salient factors important in specific geographic and economic contexts. Theory pertaining to how the characteristics of individuals may drive their perception of risks is revealed through a substantial set of quantitative psychometric studies, while theory pertaining to how the characteristics of the risks themselves drive perception is the subject of a smaller body of qualitative studies. Activities and social roles, power and trust differentials, and their interplay, all contribute to gender differences; however, we are cautioned not to equate differences in risk perception with differences in risk exposure due to an overfocus on lived experience.^7^ The meaning and value that individuals ascribe to particular risks and payoffs, while associated with gender in some cases, is additionally found to be associated with perception.^17^, ^18^ Also, a coherent body of literature has developed around the importance of risk attitudes and social preferences, as well as worldview, in shaping perception.^19^, ^20^ The objective for research such as ours is to further refine and build on the modest existing theoretical basis pertaining to gender differences in risk perception among pastoralists in low‐income countries^1^, ^2^, ^3^ by extending to smallholder farmers, by encompassing additional factors found to associate with perception in other contexts, and with an appreciation of sampling and data collection approaches that support statistical robustness and generalizability.

In a review article focusing largely on the United States and Europe, Gustafson^7^ notes that quantitative approaches that query individual perceptions about defined risks, and qualitative studies in which individuals are asked open‐ended questions about what concerns them, tend to produce disparate findings. Quantitative research consistently finds that men worry less intensely than women about nearly all of the risks studied; however, when asked to rank sets of risks men and women give strikingly similar responses. Cutter *et al*.^21^ in particular seek to distill a signal of gender‐specific differences in the ranking of risk using quantitative approaches by removing “gender‐specific” risks from consideration (i.e., those that one gender has substantially more experience with than the other).^21^ But this work does not uncover such a signal, and thus concludes that psychometric studies yield an incomplete understanding of differences by gender.

Gustafson also reviews the literature on qualitative approaches. This research consistently highlights gender differences in what individuals report worrying about in terms of relative concern, in contrast with the results of the quantitative studies. These differences are attributed to the importance of activities, roles, occupation, and/or unequal power relations.^4^, ^5^, ^6^, ^22^ For example, in Sweden, women express more concern about risks affecting home and family well‐being, whereas men express more concern with working life issues such as labor force participation.^5^ An interesting divergence of findings exists around gender differences in perception of environmental risks in the United States and Europe. Davidson and Freudenburg^6^ suggest that women's roles as nurturers and caregivers naturally orient them toward health and safety issues. Fischer *et al*.,^22^ however, find that women more frequently mention concern about environmental risks, whereas men are more concerned with health and safety risks.

More recent research has shown that men and women, on average, have different risk attitudes and social preferences and may therefore hold different perceptions regarding risky opportunities.^23^, ^24^, ^25^, ^26^, ^27^, ^28^, ^29^ Croson and Gneezy^26^ reviewed this literature and found that women tend to be more risk averse than men, that social preferences of women are more situationally specific (i.e., more malleable) than those of men, and that women are more averse to competition than men, a result confirmed by the work of Fletschner *et al*.^27^ Other recent work notes the relatively small size of gender‐associated effects and the importance of comparing at the individual level when drawing conclusions about associations.^30^

The literature exploring perceptions of risk across domains, including financial/economic, social, health/safety, occupational, ethical, environmental, war/conflict, and technology, offers explanations for observed inconsistency across gender.^19^, ^20^, ^31^ Explanatory factors revealed by earlier studies include sociocultural norms and worldview characteristics like socialism and collectivism,^18^ cultural background,^32^ and/or occupation.^31^ The cross‐domain literature also builds on the finding that individual perceptions are informed by underlying emotions associated with risk. Loewenstein *et al*.^33^ developed a “risk as feeling” approach, analogous to the Slovic *et al*.^17^ “affect heuristic,” which enables individuals to have fast, intuitive reactions to complex risk situations. This work reinforces the findings of Harshman and Paivio,^34^ who suggest that because women on average experience emotions more strongly than men, the utility of risky choices may be more heavily weighted toward extremes and risk aversion. And further, the cross‐domain work interacts with research identifying the pivotal role of the perceived benefit or cost associated with a particular risk.^35^, ^36^ Reinforcing Shoemaker's result, Weber *et al*.^20^ use a psychometric scale to assess risk attitudes by gender across domains. They conclude that women are generally more risk averse than men with respect to financial, health/safety, recreational, and ethical risks but less averse to social risks. Weber's work advances earlier statements that gender differences in perception may be less important than how the genders attach meaning to the risks, as it is not necessary to perceive risk differently to respond differently.^7^

Worldview has a rich and complex relationship with risk perception. Wildavsky and Dake^19^ were among the first researchers to suggest that cultural biases may be valuable predictors of risk perception. Four distinct worldviews are described within the cultural bias literature—hierarchical, egalitarian, fatalistic, and individualistic. Researchers have long been challenged to develop a parsimonious set of questions with which to partition individuals into these worldview categories however. An association between cultural bias and perception of certain types of risk was confirmed by Marris *et al*.^37^ though this group and others have found it difficult to categorize individuals outside of the United States and Europe according to worldview.

The existing body of research pertaining to rural low‐income contexts highlights the disconnect between perceived homogeneity in experience, livelihood, and situational conditions of resident subpopulations in contrast with a revealed heterogeneity in risk perception.^1^, ^2^, ^3^ Hofstede^38^ suggests that collectivist societies (such as those of smallholder farmers) are less risk averse because individuals have the backup of a supportive social network, and that this explains differences across countries more effectively than risk situational variables can. The more recent qualitative studies of pastoral and agro‐pastoral subpopulations in East Africa tackle heterogeneous perceptions of risk across a narrow set of domains, including drought, management of livestock, conflict/vulnerability, and human disease.^1^, ^2^, ^3^ In these studies, opportunistic open‐ended interviewing of single gender groups from Ethiopia, Kenya, and Tanzania elicits the identification and ranking of hazards. Smith *et al*.^1^ note the importance of men's and women's roles in gender differences in risk perceptions in Kenya and Ethiopia, finding that men are responsible for livestock grazing and marketing, while women are responsible for food procurement and preparation. In this context food and water availability are mentioned by approximately half of the groups regardless of gender, while risks related to gender roles, for example, animal disease and livestock prices, are expressed at different frequencies by men and women. Smith *et al*.^1^ also report extreme heterogeneity of risk responses, complicating the analysis. For most of the risks mentioned by at least one group, few other groups shared the concern, precluding an assessment of gender differences for such risks as human disease, crop impacts, farm inputs, and pasture availability.

Using the same data set, Barrett *et al*.^2^ move beyond the qualitative analysis, transforming ranks to pseudo‐cardinal index values prior to a multivariate regression in which risk perception is predicted by occupation (pastoralist, agro‐pastoralist, etc.), gender, distance from town, and relative wealth. As in Smith *et al*.,^1^ women appear to worry less about livestock‐related risks; however, sample size limits analytic power. Also, the transformation of rankings introduces an assumption that people each worry an equal “amount” overall, precluding an accounting of overall intensity of risk perception by gender. Using the same method, Quinn *et al*.^3^ explore perceptions about five sources of risk—natural, physical, financial, human, and social—in Tanzania. They confirm the importance of gender‐based roles, with men more likely to perceive risks to natural capital (e.g., land, weather, pests, livestock), while women are more likely to perceive risks to human and social capital (food, disease). Interestingly, both genders express similar perceptions of financial risk, although it is not mentioned frequently by women or men. Sample size for this analysis does not permit an isolation of the predictive power of gender.

More recently, other researchers have begun to identify gender differences related to risk perception, attitudes, and behavior in low‐income rural contexts. Charness and Gneezy^28^ analyze 15 sets of experimental data from around the world and find that women invest less and appear to be more financially risk averse than men. Cullen and Anderson^10^ analyze data collected in rural Vietnam to explore perceptions toward climate risk as it relates to farmer livelihood issues. They find that married couples sharing a household are more likely to express concordant views of risk than simulated couples consisting of a random man and a random woman selected from the population, and also that a higher proportion of male farmers report perceptions of climate change and extremes than women. Interestingly, this latter result is consistent with the finding of Quinn *et al*.^3^ that women express less concern about extreme weather than men.

We contribute to this literature both a deeper understanding of the groupings of risks into those that elicit similar (or different) individual perceptions among rural smallholder farmers in Mali, and also a distillation of the role of gender, while controlling for the broad range of other factors that represent the major theoretical bases of risk perception differences. Addressing the former, we partition the portion of variance in risk perception that is related to individual differences in overall tendency to perceive risk more intensely, from the portion that distinguishes differences between specific risks on average, using principal component analysis. Addressing the latter, we isolate the role of gender across a set of six risk domains of interest based on earlier work, while controlling for demographic (age, education, health, wealth, time poverty, number of children, geographic region) and attitudinal (social orientation, access to information, worldview, optimism, and beliefs about self‐efficacy) characteristics. Some of these covariates have been the subject of substantial previous work, others are newly included; however, controlling for this full set of factors in order to distill the role of gender has never before been possible in a rural smallholder farming context. In addition, we benefit from a larger sample than other researchers who have explored related topics and also from individual‐level responses for degree or intensity of worry about a fixed set of risks, allowing a robust statistical treatment. In contrast to research relying on rank data, our analysis benefits from a direct accounting of individual tendency to perceive risk, separate from domain‐specific perceptions. In other words we are free from assumptions that each individual is simply partitioning a fixed intensity of concern (or risk perception) qualitatively, that is, ranking a self‐generated and variably sized set of risks during enumeration. Further, our data set of individual responses collected by enumerators guards against the possibility that group dynamics will lead to the inclusion or exclusion of risks that are salient, but are perhaps less socially acceptable to discuss in a group setting. Our research also strives to expand the information base to the subpopulation of smallholder farmers, given that the focus of earlier studies was pastoralists. Gaining a better understanding of the role of gender in risk perceptions related to livelihood, as well as the relative importance of an individual's overall tendency to perceive risk, is an important step in refining the design of effective development programs that ask individuals to make changes, and in essence to assume countervailing risks, while addressing primary ones.

## 3. METHODS

### 3.1. Data

The data for our study derive from an in‐depth 2010 survey based on a stratified random sample of 1,414 single‐headed and dual‐headed households (2,703 total individual observations) across six regions in Mali (Gao, Kayes, Koulikoro, Mopti, Segou, and Sikasso). All regions in the semiarid southern part of the country (with the exception of the urban Bamako capital region) were included in the survey. Gao is the only region in our survey that is located in the northern desert—Tombouctou and Kidal were not included. The restriction of the sample to mostly southern regions is a result of long‐term unrest and conflict in the north and in northern areas of the Niger River. Of the approximately 90% of respondents who chose to self‐identify, about half were concentrated in three ethnic groups—Bambara (30.8%), Peul (10.5%), and Marka (10.5%), with the remaining respondents distributed among 15 other ethnicities, a dispersion precluding further analysis of the role of ethnicity. The survey targeted “smallholders,” defined as farmers holding less than 20 acres, the vast majority (93%) of whom rely on farming or farm labor as their main source of income. The major crops grown by these households include maize, millet, rice, sorghum, and groundnuts, with every household growing at least two of these. There was considerable variability in the overall crop portfolios by household, however, with more than 20 other crops grown by at least one household, including many vegetables.

Local enumerators administered a standard questionnaire to gather household characteristics including demographic information on all household members (Table I) and attitudes on risk and intrahousehold decision making (Table II). A total of 1,289 households had husband–wife pairs, both of whom were interviewed separately by enumerators of the same sex. In the case of polygamous households, the husband and wife with the most decision‐making authority were interviewed (i.e., head of household and most senior wife). Male heads of household answered all questions in the survey, while female respondents answered a subset of personal information, attitude, and opinion questions. The remaining households contained single occupants (101 male and 24 female respondents) who were either unmarried, widowed, divorced, or the other spouse was absent.

**Table I:** Individual and Household Demographic Characteristics

	Male	Female	Total
Gender	51.42%	48.58%	–
Age (years)	51.3 (13.9)	39.3 (12.4)	45.5 (14.5)
Children under age 15 in HH (#)	2.5 (2.2)	2.4 (2.2)	2.4 (2.2)
Income secure (self‐assessed)^a^			
No (0)	30.2%	34.6%	32.1%
Yes (1)	69.9%	65.4%	67.9%
Time poverty (self‐assessed)^b^			
Relative preference for income (0)	65.2%	64.6%	64.9%
Relative preference for time (1)	34.8%	35.4%	35.1%
Level of education			
None (0)	64.0%	82.4%	72.9%
1 or more years of education (1)	36.0%	17.6%	27.1%
Health (self‐assessed)			
Very poor (1)	0.4%	0.3%	0.3%
Poor (2)	3.4%	3.5%	3.4%
Sometimes good/sometimes poor (3)	23.9%	19.6%	22.0%
Good (4)	50.3%	57.1%	53.4%
Very good (5)	22.1%	19.6%	21.0%
Number of large livestock owned by household	2.8 (1.9)	2.4 (2.1)	2.6 (2.0)
Number of fowl and beehives owned by household	0.7 (0.8)	0.6 (08)	0.6 (0.8)
Number of crops grown/sold/consumed by household	3.7 (2.1)	3.3 (2.4)	3.5 (2.2)

^a^Survey Question: Do you feel that the income that is produced by your farm is completely adequate, nearly adequate, or not adequate to your needs? Responses are coded as “0” for no, that is, not adequate for your needs, and “1” for yes, that is, nearly adequate or adequate.

^b^Survey Question: If you had a choice, which of the following would you prefer. Please rank them in order of 1 = most preferred, 2 = second most preferred, and 3 = third most preferred: Work the same number of hours daily but have a little more food and income; Work a few more hours daily but have a lot more food and income; Work fewer hours daily but be able to maintain my current level of food and income. Responses are coded as “0” if “Work a few more hours daily but have a lot more food and income” is ranked higher than “Work fewer hours daily but be able to maintain my current level of food and income” and “1” if the opposite is true.

**Table II:** Individual Personality Traits

	Male	Female	Total
Extroversion: “When I find a new farm method that I really like, I have to tell others about it.”			
Disagree completely (1)	2.12%	2.22%	2.16%
Somewhat disagree (2)	7.23%	9.05%	8.05%
Neither agree nor disagree (3)	28.69%	34.78%	31.44%
Somewhat agree (4)	37.23%	34.61%	36.04%
Agree completely (5)	24.74%	19.34%	22.31%
Fatalistic: “If misfortune is meant to strike my farm I cannot avoid it.”			
Disagree completely (1)	7.37%	6.92%	7.17%
Somewhat disagree (2)	13.87%	16.33%	14.98%
Neither agree nor disagree (3)	35.62%	39.57%	37.40%
Somewhat agree (4)	24.89%	23.25%	24.15%
Agree completely (5)	18.25%	13.93%	16.30%
Individualistic: “I do not want anyone to tell me what to do on my farm.”			
Disagree completely (1)	21.24%	15.44%	18.62%
Somewhat disagree (2)	27.96%	29.90%	28.83%
Neither agree nor disagree (3)	22.85%	26.89%	24.67%
Somewhat agree (4)	17.66%	18.99%	18.26%
Agree completely (5)	10.29%	8.78%	9.61%
Attitude toward the future: “How would you describe your attitude toward the future?”			
Very/somewhat pessimistic (0)	12.93%	12.94%	12.94%
Very/somewhat optimistic (1)	87.07%	87.06%	87.06%
Risk tolerance			
“Prefer a job that is lower in income but continuous and assured” (0)	90.15%	91.67%	90.83%
“Prefer a job that is higher on income but can be lost anytime” (1)	9.85%	8.33%	9.17%
Self‐efficacy: “How confident are you that you can learn how to do this activity?”			
Not confident (1)	6.26%	8.89%	7.45%
Somewhat confident (2)	35.05%	45.42%	39.75%
Very confident (3)	58.69%	45.69%	52.80%
Social: “I often discuss farming methods and issues with other people.”			
Disagree completely (1)	2.70%	2.22%	2.48%
Somewhat disagree (2)	8.69%	10.74%	9.61%
Neither agree nor disagree (3)	28.61%	37.53%	32.64%
Somewhat agree (4)	36.72%	35.23%	36.04%
Agree completely (5)	23.28%	14.29%	19.22%
Network: # of information sources contacted by mobile phone			
(0)	68.20%	84.39%	76.06%
(1)	16.19%	11.88%	14.10%
(2)	12.01%	3.27%	7.77%
(3)	3.02%	0.46%	1.78%
(4)	0.36%	–	0.18%
(5)	0.22%	–	0.11%

**Figure 1:**
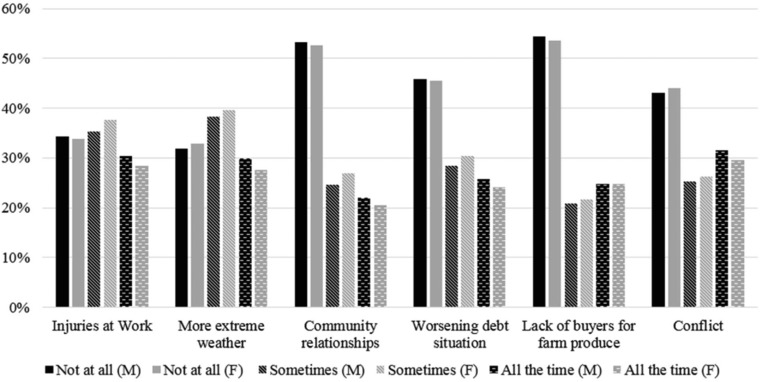
Level of concern (perceived risk) by gender across six risk domains, as expressed in answer to the question: How frequently have you worried about the following risks in the past 12 months? Not at all; Sometimes; All the time.

The objectives of the survey were to understand how farmers could be segmented for agricultural extension based on their perceptions of the risks and opportunities they face, their psychometric characteristics, their worldview, and their motivations, in order to improve development interventions. To address this objective the survey included questions about risk perceptions for six risk domains: injuries at work; climate and extreme weather; community relationships; debt; livelihoods; and large‐scale conflict. Each respondent was asked to assess their level of concern about each risk domain by selecting one of three worry levels (Fig. 1). In our analysis these self‐reported levels of worry are treated as proxies for level of perceived risk. The question was worded as follows:

*How frequently have you worried about the following risks in the past 12 months? (Read/show card of options): (1) Not at all; (2) Sometimes; (3) All the time*.

- Injuries at work
- More extreme weather—example—hot spells
- Community relationships
- Worsening debt
- Lack of buyers (for farm produce)
- Conflict

Table II summarizes variables used to proxy for individual personality traits, including worldview, risk preferences, self‐efficacy, and access to social networks. We include three survey questions designed to cut across multiple dimensions of worldview, including fatalism, individualism, and social orientations. Fatalism is gauged by the extent to which individuals agreed with the prompt: “*If misfortune is meant to strike my farm I can not avoid it*.” Individualism is reflected by an individual's level of agreement with the prompt: “*I do not like anyone to tell me what to do on my farm*.” A social orientation is assessed by an individual's level of agreement with the prompt: “*I often discuss farming issues and methods with others*.” Risk tolerance is self‐assessed based on the prompt: “*Which of the following do you prefer*?” with two possible responses “*A job that is higher on income but can be lost anytime*” (coded as risk tolerant), and “*A job that is lower on income but it is continuous and assured*” (coded as risk averse). Optimism is self‐assessed with the prompt “*What is your attitude about the future?*” with four response categories: “*very optimistic*,” “*somewhat optimistic*,” “*somewhat pessimistic*,” and “*very pessimistic*.” Optimism is then recoded to a binary form such that “somewhat” and “very” optimistic are grouped together as “optimism” and “somewhat” or “very” pessimistic are grouped as “pessimism.” We proxy “extroversion” as an individual personality trait by asking respondents the degree to which they agree with the prompt: “*When I find a new farm method that I really like I have to tell others about it*.” The strength of a respondent's network connectedness is represented by the number of information sources he/she uses a mobile device to access.

### 3.2. Analysis

We follow Weber *et al*.^20^ in looking across domains while gauging the role of gender and other predictors in risk perception. We carry out dimensional reduction analysis (principal component analysis) in order to resolve the variability structure in responses across six risk domains. Specifically, we use principal component analysis to identify common modes of risk perception expressed as worry, and to understand which risk domains tend to elicit similar responses in survey respondents.

We apply multiple regression analysis to gauge the importance of individual predictors including gender, age, education, self‐reported health, presence of children in the home, income security, time poverty, self‐assessed health, worldview, optimism about the future, risk orientation, self‐efficacy, access to information, livestock holdings, and diversity of agricultural portfolio, related to an individual's answer to the question: “Do you worry about X?” Our use of categorical outcome variables for the primary OLS regression analysis is supported by a robust distribution of responses across the three possible answers for both male and female respondents, that is, “All the time,” “Sometimes,” and “Not at all.”

Separately, we probe the importance of geographic region in the analyses outlined above. We generated an individual principal component analysis for each region, and also a regression analysis in which region is controlled with a set of dummy variables. For the regression Segou serves as the reference region given its central location among our surveyed areas.

Finally, we tested the stability and robustness of our findings using alternative model specifications with the dependent variable aggregated into a binary 1/0 outcome. For those individuals answering the question “How often do you worry about risk X?” with “Not at all” we assigned a value of 0, and for those answering “Sometimes” or “All the time” we assigned a 1. This approach is helpful for probing differences that may be attributable to style of communication rather than risk perception. We are aware that individuals who worry about a particular risk may distinguish less between worrying all the time and some of the time, as compared to never worrying. To address this issue we ran both OLS and logit regressions with the binary form outcome variable, controlling for the same predictors as in our primary regression.

## 4. RESULTS

We observe somewhat higher levels of worry for both genders for work injury, extreme weather, and conflict, and the differential between male and female responses is also slightly larger for these domains. The level of worry varies with gender as women are more likely to report worrying “Sometimes” in all domains while men are more likely to report worrying “All the time,” for all but the risk of a lack of buyers (Fig. 1 and Appendix).

**Figure 2:**
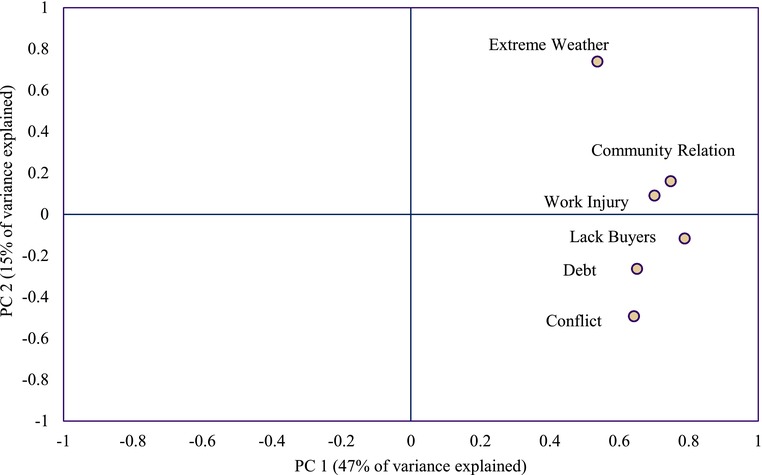
Principal component analysis score plot for individual expressions of risk perception for six risk domains. PC1 explains 47% of overall variability, PC2 explains 15% of overall variability.

Principal component analysis (PCA) distills patterns in individual profiles of risk perception across the six domains of risk (Fig. 2). Note that risk domains with similar scores for the principal components (i.e., domains that appear in close proximity in a scatter plot of PC1 vs. PC2) can be interpreted as eliciting similar responses among individuals. In the first principal component (PC1) the risk domains are very tightly clustered along the *x*‐axis, and this factor explains about 47% of the overall variability in responses (Fig. 2). PC1 may be interpreted as distilling the portion of overall variance attributable to the presence of individuals who are more inclined to perceive risk or worry across domains, versus those less inclined. In the scores for the second component (PC2), however, there is much more variability, and this component explains only about 15% of overall variance in risk perception patterns. Worry associated with the strength and health of community relationships and work injury appear clustered in the score plot, indicating that on average there are some similarities in individual patterns of response about these risks to emotional and physical health. Meanwhile, lack of buyers at the market and debt, both sources of worry related to an individual's financial situation, plot in fairly close proximity. The domains that are most distinct are conflict, with the most negative score in PC2, and more extreme weather, with the highest positive score in PC2. These results suggest a qualitative difference between concern about extreme weather and conflict (risks that are expected to vary in intensity by geographic region and are largely beyond the control of individuals) and concern about the other domains, which are expected to be more universally experienced across the country, more familiar, or more closely tied to individual choices and behaviors.

The potential role of regional location, especially relative to the importance of concerns about extreme weather and conflict in explaining variance in individual response patterns, warrants additional exploration. Thus, we carry out regional PCA analyses (available as Supplementary Material). Our sample size is adequate to support individual analysis for all regions except Gao, where small sample size and lack of variance preclude this treatment. The regional PCA analyses reveal clustering and patterns similar to the aggregate analysis. In three of the five regions (Segou, Kayes, and Koulikoro), extreme weather and conflict are at the extremes of PC2, identical to the aggregate results. In Mopti, concern about debt holds the extreme in PC2, with the remaining risk positions conforming to the aggregate results, while in Sikasso, debt and community relationships are at the extremes.

Categorical OLS regression models predicting frequency of worry for each domain as a function of demographic, attitudinal, and household characteristics explain between 8% and 17% of the outcome variance (Table III) in countrywide analysis. The highest adjusted *R* ^2^ values are associated with risk to community relationships (15%) and conflict (17%), while the lowest is for extreme weather (8%). Being female is associated with higher risk perception for all domains except extreme weather, and gender is a statistically significant predictor of worry for all queried risk domains. The largest gender effect is revealed in association with lack of buyers (which is a livelihood risk) while the gender effect for other risks is relatively small. A respondent's age is not generally a significant predictor of risk perception, except for conflict, where being older is associated with more frequent worry, and community relationships, where being older is associated with less frequent worry. The presence of children under the age of 15 in the household has a significant association with less frequent worry about work injury, extreme weather, and community relationships.

**Table III:** Categorical OLS Analysis Outcome Variable: How Often Do You Worry about [Risk X]? 1 = *Not at All*; 2 = *Sometimes*; 3 = *All the Time*

	(1)Work Injury	(2)Extreme Weather	(3)Community Relation	(4)Debt	(5)Lack of Buyers	(6) Conflict
Sex (male = 0; female = 1)	0.069*	−0.065*	0.070*	0.069*	0.161***	0.087*
	(0.036)	(0.036)	(0.035)	(0.037)	(0.038)	(0.037)
Age (years)	0.001	−0.001	−0.002*	−0.000	0.002	0.005***
	(0.001)	(0.001)	(0.001)	(0.001)	(0.001)	(0.001)
Children under age 15 (# in household)	−0.024***	−0.018*	−0.024**	0.012	−0.007	0.008
	(0.007)	(0.007)	(0.007)	(0.008)	(0.008)	(0.008)
Income secure (0 = no; 1 = yes)	−0.218***	−0.154***	0.001	0.084*	0.136***	−0.056
	(0.034)	(0.035)	(0.034)	(0.035)	(0.036)	(0.036)
Time poverty	0.080*	0.099**	−0.004	−0.176***	0.014	0.053
(0 = preference for income; 1 = preference for time)	(0.033)	(0.034)	(0.033)	(0.034)	(0.035)	(0.035)
Education level (0 = none; 1 = 1 or more years)	0.060	0.007	0.081*	0.071*	0.093*	0.054
	(0.037)	(0.037)	(0.036)	(0.038)	(0.039)	(0.038)
Health (1 = very poor to 5 = very good)	0.058**	0.024	0.033	−0.004	0.082***	0.060**
	(0.021)	(0.022)	(0.021)	(0.022)	(0.023)	(0.022)
Fatalistic	0.070***	0.031*	0.091***	0.039**	0.017	0.133***
	(0.014)	(0.014)	(0.014)	(0.015)	(0.015)	(0.015)
Individualistic	0.040**	0.056***	0.109***	0.090***	0.104***	0.069***
	(0.013)	(0.013)	(0.013)	(0.013)	(0.013)	(0.013)
Socially oriented	0.030*	0.002	0.049**	0.046*	0.055**	0.118***
	(0.018)	(0.018)	(0.018)	(0.018)	(0.019)	(0.019)
Attitude toward the future	−0.131**	−0.127**	−0.160***	−0.126**	−0.235***	−0.063
(0 = pessimism; 1 = optimism)	(0.046)	(0.047)	(0.046)	(0.048)	(0.049)	(0.048)
Risk tolerance (0 = no, 1 = yes)	−0.295***	−0.227***	−0.206***	−0.122*	−0.204***	−0.348***
	(0.054)	(0.054)	(0.053)	(0.056)	(0.057)	(0.056)
Self‐efficacy	0.101***	0.062*	0.065**	0.029	0.055*	0.034
(1 = not confident to 3 = very confident)	(0.025)	(0.026)	(0.025)	(0.026)	(0.027)	(0.027)
Extroversion	0.085***	−0.053**	0.066***	0.048**	0.113***	0.118***
	(0.017)	(0.017)	(0.017)	(0.018)	(0.018)	(0.018)
Network (# of info sources contacted by mobile)	0.148***	0.101***	0.227***	0.233***	0.216***	0.163***
	(0.021)	(0.022)	(0.021)	(0.022)	(0.023)	(0.022)
Large livestock (#)	−0.024**	−0.014	−0.018*	−0.014	−0.013	−0.001
	(0.009)	(0.009)	(0.009)	(0.009)	(0.009)	(0.009)
Fowl and beehives (#)	−0.087***	−0.078***	−0.097***	−0.028	−0.049*	−0.017
	(0.021)	(0.021)	(0.021)	(0.022)	(0.022)	(0.022)
Crop portfolio diversity (# grown/sold/consumed)	−0.008	−0.043***	−0.013*	0.012	−0.011	0.040***
	(0.008)	(0.008)	(0.008)	(0.008)	(0.008)	(0.008)
Constant	1.026***	2.184***	0.732***	1.003***	0.296*	−0.350*
	(0.158)	(0.161)	(0.157)	(0.164)	(0.168)	(0.166)
Observations	2,383	2,370	2,415	2,393	2,386	2,402
Adjusted *R* ^2^	0.12	0.08	0.15	0.10	0.12	0.17

*Note*: Standard error in parentheses.

^*^ *p* < 0.1, ^**^ *p* < 0.01, ^***^ *p* < 0.001.

Income security and time poverty (i.e., a stated preference for more free or leisure time as opposed to a preference for more income), demonstrate related impacts on risk perception. Income security is associated with less frequent worry about work injury and extreme weather, while relative time poverty is associated with more frequent worry for work injury and extreme weather. By contrast, income security is associated with more frequent worry about debt and lack of buyers, while time poverty is associated with less frequent worry about debt.

Education and self‐assessed health do not show a uniform pattern as predictors of risk perception. Having obtained at least one year of education is associated with more frequent worry across all risk domains, with statistically significant associations for community relationships, debt, and a lack of buyers. Self‐assessed better health is associated with higher perception of risk of work injury, lack of buyers, and conflict, and not associated with the other three risks.

We observe the importance of worldview predictors with perception of risk across the six domains. Individuals with a fatalistic worldview express more frequent worry about five of the six risk domains, with high levels of significance (the exception is lack of buyers). Those expressing an individualistic worldview report significantly more frequent worry across all six domains. Finally, individuals who express a social orientation also express significantly more frequent worry about all risk domains with the exception of extreme weather, where the association is also positive but lacks significance.

Extroversion, optimism about the future, risk orientation, and self‐efficacy also reveal important associations with risk perception. Extroverts express significantly more frequent worry, except in the case of extreme weather where the association is with less frequent worry. Optimists express less frequent worry across all domains except conflict, while risk seekers worry less across all domains. The latter two predictors are associated with the largest effects overall. On the other hand, self‐efficacy is associated with significantly more frequent worry about work injury, extreme weather, community relationships, and lack of buyers.

The final set of predictors includes “network” (which represents the number of information sources one uses a mobile device to access), livestock holdings, and also the diversity of a household's agricultural portfolio. We observe a strong association between an individual's access to more information sources and more frequent worry across all risk domains, with relatively large effect sizes. Individuals reporting more livestock holdings express less frequent worry about work injury and community relationships. Individuals reporting owning more fowl and beehives report significantly less frequent worry about all domains except debt and conflict. Finally, individuals with more diverse crop portfolios report less frequent worry about extreme weather and community relationships, but more worry about conflict.

We compare the results for Mali as a whole (Table III) with those that include each individual's region of residence as a predictor (Table IV). The direction of the effect of gender as a predictor remains unchanged across all domains; however, it is no longer statistically significant in the models for extreme weather, conflict, and work injury, while for lack of buyers gender remains significant at *p* < 0.01. Relative to the reference region Segou, residence in Gao is associated with less frequent worry about extreme weather and debt, and residence in Kayes, Koulikoro, and Sikasso is associated with less worry for nearly all risk domains, the exceptions being debt (more frequent worry in Koulikoro, not significant for Sikasso) and extreme weather (more frequent worry for Kayes, not significant for Koulikoro). Residence in Mopti is associated with less frequent worry than in Segou for work injury, lack of buyers, and conflict. With the addition of regional control variables the statistical significance of other predictors changes relative to the models without region in some cases, but in only one instance is the effect direction changed (i.e., for income security, which is a predictor of *less frequent worry* about community relationships in the model with regions). Overall, with the introduction of regional controls, worry about conflict, lack of buyers, and debt are the only risk domains whose models gain more than a few percent in explanatory power (measured as adjusted *R* ^2^). In the model for conflict, we observe that income security and fowl/bee holdings are now negatively associated with worry, while time poverty, self‐efficacy, and as livestock holdings are positively associated. In the model for lack of buyers, time poverty picks up significance for a positive association, while income security, education, and risk tolerance lose significance. Finally, in the model for debt, income security, education, risk tolerance, and extroversion lose significance while self‐efficacy picks up a positive association, and ownership of fowl and beehives picks up a negative association.

**Table IV:** Categorical OLS Analysis (Controlled for Region) Outcome Variable: How Often Do You Worry about [Risk X]? 1 = *Not at All*; 2 = *Sometimes*; 3 = *All the Time*

	(1)Work Injury	(2)Extreme Weather	(3)Community Relation	(4)Debt	(5)Lack of Buyers	(6) Conflict
Sex (male = 0; female = 1)	0.047	−0.056	0.063*	0.074*	0.133***	0.056
	(0.035)	(0.036)	(0.035)	(0.036)	(0.036)	(0.035)
Age (years)	0.001	−0.001	−0.002*	0.001	0.002	0.005***
	(0.001)	(0.001)	(0.001)	(0.001)	(0.001)	(0.001)
Children under age 15 (# in household)	−0.027***	−0.014*	−0.025***	0.006	−0.013*	0.005
	(0.007)	(0.007)	(0.007)	(0.007)	(0.007)	(0.007)
Income secure (0 = no; 1 = yes)	−0.245***	−0.120***	−0.076*	0.021	0.034	−0.142***
	(0.035)	(0.036)	(0.035)	(0.036)	(0.036)	(0.035)
Time poverty	0.107**	0.048	0.043	−0.067*	0.100**	0.125***
(0 = preference for income; 1 = preference for time)	(0.034)	(0.035)	(0.034)	(0.035)	(0.035)	(0.034)
Education level (0 = none; 1 = 1 or more years)	0.038	−0.004	0.041	0.047	0.052	−0.002
	(0.036)	(0.037)	(0.036)	(0.037)	(0.037)	(0.036)
Self‐health assessment (1 = very poor to 5 = very good)	0.065**	0.027	0.017	−0.003	0.071**	0.062**
	(0.021)	(0.022)	(0.021)	(0.022)	(0.022)	(0.021)
Fatalistic	0.053***	0.027*	0.061***	0.026*	−0.013	0.088***
	(0.014)	(0.015)	(0.014)	(0.015)	(0.015)	(0.014)
Individualistic	0.047***	0.054***	0.093***	0.074***	0.104***	0.064***
	(0.013)	(0.013)	(0.013)	(0.013)	(0.013)	(0.013)
Socially oriented	0.037*	0.023	0.053**	0.053**	0.059**	0.128***
	(0.018)	(0.019)	(0.018)	(0.018)	(0.019)	(0.018)
Attitude toward the future (0 = pessimism; 1 = optimism)	−0.087*	−0.127**	−0.117**	−0.083*	−0.162***	0.020
	(0.045)	(0.046)	(0.045)	(0.046)	(0.047)	(0.045)
Risk tolerance (0 = no; 1 = yes)	−0.220***	−0.220***	−0.110*	−0.045	−0.079	−0.172**
	(0.054)	(0.055)	(0.053)	(0.055)	(0.055)	(0.053)
Self‐efficacy (1, 2, 3)	0.121***	0.060*	0.067**	0.082**	0.069*	0.075**
	(0.026)	(0.027)	(0.026)	(0.027)	(0.027)	(0.026)
Extroversion	0.066***	−0.023	0.039*	0.016	0.064***	0.074***
	(0.018)	(0.018)	(0.017)	(0.018)	(0.018)	(0.017)
Network (# of info sources contacted by mobile)	0.083***	0.113***	0.173***	0.152***	0.110***	0.043*
	(0.022)	(0.023)	(0.022)	(0.022)	(0.023)	(0.022)
Large livestock (#)	−0.016*	−0.024**	−0.011	0.003	−0.000	0.017*
	(0.009)	(0.009)	(0.009)	(0.009)	(0.009)	(0.009)
Fowl and beehives (#)	−0.113***	−0.041*	−0.129***	−0.063**	−0.109***	−0.074***
	(0.022)	(0.022)	(0.021)	(0.022)	(0.022)	(0.022)
Crop portfolio diversity (# grown/sold/consumed)	−0.014*	−0.035***	−0.008	0.002	−0.013	0.033***
	(0.008)	(0.008)	(0.008)	(0.008)	(0.008)	(0.008)
Segou (reference region)	0.000	0.000	0.000	0.000	0.000	0.000
	(.)	(.)	(.)	(.)	(.)	(.)
Gao	0.055	0.219**	0.033	0.189*	0.083	0.046
	(0.076)	(0.077)	(0.075)	(0.078)	(0.078)	(0.075)
Kayes	−0.417***	0.213***	−0.409***	−0.436***	−0.758***	−0.827***
	(0.054)	(0.055)	(0.053)	(0.055)	(0.056)	(0.054)
Koulikoro	−0.118*	0.034	−0.123*	0.138*	−0.239***	−0.179***
	(0.054)	(0.055)	(0.053)	(0.055)	(0.055)	(0.053)
Mopti	−0.282***	−0.014	0.044	0.055	−0.210***	−0.329***
	(0.054)	(0.056)	(0.054)	(0.055)	(0.056)	(0.054)
Sikasso	−0.311***	−0.221**	−0.371***	−0.008	−0.397***	−0.684***
	(0.074)	(0.076)	(0.070)	(0.075)	(0.077)	(0.072)
Constant	1.255***	1.896***	1.180***	1.079***	0.943***	0.215
	(0.185)	(0.190)	(0.183)	(0.189)	(0.191)	(0.184)
Observations	2,383	2,370	2,415	2,393	2,386	2,402
Adjusted *R* ^2^	0.16	0.09	0.18	0.16	0.21	0.28

*Note*: Standard errors in parentheses.

^*^ *p* < 0.1, ^**^ *p* < 0.01, ^***^ *p* < 0.001.

Binary OLS and logit regression analyses (i.e., recoding the three risk perception categories of response to a binary outcome variable), carried out as robustness checks, yield very similar results to the categorical OLS described in Tables III and IV.

## 5. DISCUSSION

This study examines the potential role of gender in shaping risk perceptions in rural Malian smallholder farm households. Controlling for individual demographic and attitudinal characteristics we distill a significant effect of gender on perceived risk in countrywide analysis, while noting similar raw levels of risk perception between males and females. While our data do not address the question of whether male and female smallholder farmers in Mali perceive risk differently because of differences in perceptions of the implications, or because of differences in perception relative to mitigation options, our aggregate findings of gender‐differentiated responses to risk for Mali as a whole are consistent with previous research.^1^, ^2^, ^3^, ^6^, ^7^ When we control for geographic region within Mali, however, the effects of some predictors change (Tables III and IV), in particular, the statistical significance of gender as a predictor disappears for worry about work injury, extreme weather, and conflict. These changes in coefficient estimates with regional controls suggest that some variation is primarily at the regional level, whereas other variation is sufficiently diffuse throughout regions to be picked up at the individual level.

The most substantial change with the introduction of region as a predictor is in the model for worry about conflict, which experiences the greatest increase in adjusted *R* ^2^ among our six risk domains, and for which predictors beyond region also come into play, for example, income security, self‐efficacy, and time poverty. Despite the exclusion of high‐conflict areas (mainly in the north) from the sample, our results suggest that conflict is a widely perceived risk in Mali, though there is spatial variation in perception even in the south. The other model with a relatively large increase in explanatory power with the addition of regional controls is for frequency of worry about lack of buyers for farm produce. As noted above, lack of buyers and debt share similar patterns of risk perception in the dimension reduction analytic results, which suggests that the financial component shared by these two risks may be influencing survey responses. Our sample in Mali is entirely smallholder farmers, and thus the financial risk associated with a lack of buyers for farm produce at the market is familiar to both males and females, and carries important impacts for both. Income security, education, and risk tolerance lose statistical significance when the lack of buyers model is controlled for region, which may reflect that buyers and markets are regionally diverse. The persistent gender signal in the model for risk of lack of buyers reinforces findings from Weber *et al*.^20^ and Charness and Gneezy,^28^ who find that women tend to be more risk averse than men in financial domains, as noted above.

Our regression and dimension reduction results reveal that although individuals clearly do not worry uniformly across domains (Fig. 1), the most important explanatory factor in the variance structure of responses is whether an individual tends to be a worrier or not (Fig. 2). The first principal component explains nearly half of the variance in responses on this basis alone. This finding expands beyond previous results from low‐income countries where data structures force an assumption that all individuals worry with equal intensity overall.^1^, ^2^, ^3^ Further, while loadings on the second principal component explain a much smaller fraction of variance in responses, they reveal the importance of qualitative differences between risks, with extreme weather and conflict (forces that vary with geographical location within the country and are largely out of the hands of individuals) plotting outside the main cluster of risks (which are more closely tied to individual choices and behaviors). When dimension reduction analysis is carried out separately for each region, we observe some differences by geographic location although the overall picture is largely maintained—with overriding importance attached to whether an individual tends to be a worrier or not, while differences across risk domains play a much smaller role in explaining patterns of individual risk perception.

In regression models, in the context of poor smallholder farmers for Mali as a whole, we find that being female is generally associated with greater perception of risk for all domains with the exception of extreme weather, where being female is associated with less concern, although when controlling for region the significance of the gender effect is largely muted. Our findings reveal a weaker gender signal than that reported by Weber *et al*.,^20^ who find women to be more risk averse across domains except with respect to personal social risk. Findings from both low‐income country contexts, as well as from the United States and Europe, suggest that women are generally more concerned about risks affecting home and family, with men more concerned about work‐related risks, and mixed results regarding risks to the environment, and to health and safety. Barrett *et al*.^2^ and Quinn *et al*.^3^ report for pastoralist subpopulations in East Africa that women express lower perception of risk across domains related to livestock, pasture, water, and weather and higher perception of risk related to violent conflict and human disease. These analyses provide a limited basis for comparison with ours as Barrett's results are controlled only for occupation, distance to town, and relative wealth, and Quinn's are not controlled. Still, where overlap exists we note consistency with our results for Mali as a whole, since men perceive more risk related to extreme weather and women perceive more risk related to conflict and human health when geographic region is not considered. Cullen and Anderson^10^ similarly observe that women in a low‐income context report lower perception of weather extremes such as heat, drought, and heavy rains.

For other demographic correlates of risk perception, we find that age is a significant predictor only for conflict, where being older is associated with higher risk perception, and community relationships, where being older is associated with lower risk perception. In future work it would be interesting to test the role of relative status within one's household, distinct from age, on risk perception. In this survey all of the respondents were male heads of household and senior, or sole, wives; thus comparisons across status were not possible. Further, while we did explore polygamy/monogamy as a predictor of risk perception in a robustness check, we found that it contributed no additional predictive power, nor was it statistically significant—likely due to a lack of variability in the sample. The association of income security with more frequent worry about debt and lack of buyers, though only significant when we do not control for geographic region, is consistent with the potential impact on income posed by these risks and their immediacy with respect to individual decisions and actions. The reverse association with extreme weather and conflict is perhaps surprising given that these risks are imposed largely externally, but still hold significant implications for earnings and income in the agricultural sector.

Worldview reveals a rich and complex relationship with risk perception that is robust whether we control for region or not. Researchers have long been challenged to develop a parsimonious set of questions with which to partition individuals into those who view the world with a lens colored by preferences for hierarchy, or community cohesion, or reliance on self, or other things. Consistent with earlier research we find that the categories of worldview are not mutually exclusive.^37^ Still, all three of our worldview questions are highly significant predictors of risk perception across risk domains. Fatalists express significantly more frequent worry about every risk domain except lack of buyers. Individualists express significantly more frequent worry across every risk domain. Those expressing a social orientation also report more frequent worry across domains, except for the risk of more extreme weather. Because the social orientation question captures a preference for communicating with others specifically about farm issues, perhaps the perception of extreme weather risk is neutralized by sharing information about compensating strategies for coping with extreme weather. The distinction we identify for extreme weather is consistent with the cushion hypothesis of Hofstede,^38^ which suggests that individuals in collectivist societies will express less risk aversion because they have backup from the group.

Our results on personality traits as risk perception predictors are consistent with past research results as well. Nicholson *et al*.^31^ find that risk taking is associated with both extroversion and the tendency for sensation seeking, though these results may be attributable more to feelings about the relative weight of the cost and benefit outcome balance, rather than a lack of concern or worry. We find that extroversion is associated with increased perception of risk across all queried risk categories except extreme weather (though this exception is not statistically significant when controlling for region), while risk tolerance is associated with decreased perception of risk. We note less concern about all categories of risk among optimists, consistent with both Nicholson *et al*.^31^ and Cullen and Anderson.^10^

Finally, we consider the possibility that our findings on gender may in part reflect gender differences in style of communication rather than a difference in risk framing and/or perception. In our raw survey responses (Fig. 1) we observe a strong pattern of men giving the response “all the time” more often than women, and women giving the response “sometimes” more often than men. However, when we construct a binary outcome variable (which aggregates the worry “all the time” and the worry “sometimes” categories) our categorical OLS regression results are reproduced robustly with both logit and binary OLS. Based on this secondary finding we believe that we are indeed examining gender differences in risk perception and attitude rather than differences in communication about risk.

## 6. CONCLUSIONS

Our results contribute to the understanding of gender‐based differences in risk perception in low‐income rural contexts and in particular among smallholder farmers in Mali. We confirm earlier findings of heterogeneity in risk perception and highlight the importance of considering variation in overall tendency to worry when interpreting risk perception results across individuals and across domains. Interestingly, we observe overall that women express that they worry “sometimes” about individual risks most frequently, while men express that they worry “all the time” most frequently. But while our regression results for Mali as a whole reveal gender differences in risk perception across domains, this is largely absent when we control for geographic region, except with regard to the risk of a lack of buyers for farm produce, where women express distinctly more concern regardless (*p* < 0.01). Our data set does not support evaluation of whether these results translate into a higher likelihood of development program uptake among women than men when financial risks are targeted. Still, we argue that it is important to consider an individual's perception of the countervailing risks of participating in an offered program, in contrast with the targeted risk. It is possible that interventions cast as risk reducing may thus be more appealing and more effective than interventions cast as income increasing.

Further, level of perceived individual control over, and level of access to information about, each risk domain matters in the perception of risk. Specifically, conflict and extreme weather are distinguished by unique patterns of participant response, perhaps as a result of being viewed as less subject to individual choice or control relative to the other risk domains. The largest predictor effects we observe are for level of access to information sources, risk orientation and geographic region. Access to information is associated with higher levels of risk perception while, by contrast, optimism about the future and risk tolerance is associated with lower levels. The strong effect of region points to variability in risk perception across Mali and its inclusion diminishes the effect size associated with information access, indicating a geographic component to availability of information sources. We note that where connection to more information sources is associated with higher or more intense perception of risk, providing individuals with additional access to information may contribute to more interest in mitigating certain risks, as these will become more salient and perhaps more worth the cost of the perceived countervailing risk of making a change.

In summary, an improved overall understanding of the drivers of men's and women's risk perceptions opens up opportunities for international development organizations to better design interventions. Programs may be tailored for individuals, and in particular should take geographic variability into account, while offering the chance to mitigate primary targeted risks by introducing changes in behaviors, activities, or practices that may be perceived as risky themselves.

## Supporting information


**Supplemental Material**

## OUTCOME VARIABLES DESCRIPTIVE STATISTICS

### 1

**Table:**

	Male		Female		Total	
	Freq.	%	Freq.	%	Freq.	%
Risk: work injury						
Not at all (1)	459	34.36%	365	33.36%	824	33.91%
Sometimes (2)	472	35.33%	444	40.59%	916	37.70%
All the time (3)	405	30.31%	285	26.05%	690	28.40%
Risk: more extreme weather						
Not at all (1)	423	31.85%	369	33.92%	792	32.78%
Sometimes (2)	508	38.25%	448	41.18%	956	39.57%
All the time (3)	397	29.89%	271	24.91%	668	27.65%
Risk: community relationships						
Not at all (1)	720	53.33%	573	51.53%	1293	52.52%
Sometimes (2)	332	24.59%	330	29.68%	662	26.89%
All the time (3)	298	22.07%	209	18.79%	507	20.59%
Risk: debt						
Not at all (1)	613	45.78%	496	45.05%	1109	45.45%
Sometimes (2)	380	28.38%	362	32.88%	742	30.41%
All the time (3)	346	25.84%	243	22.07%	589	24.14%
Risk: lack of buyers						
Not at all (1)	729	54.48%	573	52.33%	1302	53.51%
Sometimes (2)	278	20.78%	250	22.83%	528	21.70%
All the time (3)	331	24.74%	272	24.84%	603	24.78%
Risk: conflict						
Not at all (1)	580	43.12%	499	45.20%	1079	44.06%
Sometimes (2)	340	25.28%	305	27.63%	645	26.34%
All the time (3)	425	31.60%	300	27.17%	725	29.60%
