# Supplementary material for: Variability in Cross‐Domain Risk Perception among Smallholder Farmers in Mali by Gender and Other Demographic and Attitudinal Characteristics
Source: Risk Anal. 2018 Feb 15;38(7):1361–77. doi: 10.1111/risa.12976 (PMC6055814; doi:10.1111/risa.12976)
Supplement: Supplementary file 1 — Supplemental Material [file RISA-38-1361-s001.docx]

**Supplemental Materials:**

**Principal Component Analyses (PCA): Individual Score Plots by Region for PC1 and PC2 for Six Risk Domains**

**Variability in Cross Domain Risk Perception among Smallholder Farmers in Mali by Gender and other Demographic and Attitudinal Characteristics**

Alison C. Cullen^1^, C. Leigh Anderson^1^, Pierre Biscaye^1^, Travis W. Reynolds^2^

^1^Daniel J. Evans School of Public Policy and Governance, University of Washington, Seattle, WA, USA.

^2^Environmental Studies Program, Colby College, Waterville, ME, USA.

∗Address Correspondence to Alison C. Cullen, Daniel J. Evans School of Public Policy and Governance, University of Washington, 208 Parrington Hall, Box 353055, Seattle, WA 98185-3055, USA; tel: +1-206 616-1654; alison@uw.edu.
